# 

**Published:** 1892-12-31

**Authors:** 


**Notices of Births, Deaths, and Marriages,** are inserted in this column at a charge of 2s. 6d. for an announcement not exceeding four lines.

### NOTICE TO CORRESPONDENTS.

All MS., Letters, Books for Review, and other matters intended for the Editor should be addressed The Editor, The Lodge, Porchester Square, London, W.

The Editor cannot undertake to return rejected M.S., even when accompanied by stamped directed envelope.

**Notice to Advertisers and Subscribers.**

All Advertisements, Orders for Copies of The Hospital, and Business Communications should be addressed to The Publisher, *not to the Editor*, The Hospital, 140, Strand, W.C.

The Cost of Subscription, post free, is as follows:—Per week, 2½d.; per quarter, 2s. 6d.; per half-year, 5s.; per annum, 8s. 6d. Foreign:—Per annum, 10s. 8d.; payable, in all cases, in advance.

**"Burdett's Hospital Annual," 1893.**

Fourth year of publication. 700 pp. Boards, gilt lettering. A most complete guide to British, Colonial, Indian, and American Hospitals, Dispensaries, Nursing and Convalescent Institutions, and Asylums. Price 5s. Published by The Scientific Press (Limited), 140, Strand, W.C.

**NOTICE.—The Index for Vol. XII. (ending September 1892) is on sale at the Office of this Journal, price 2½d., post free.**

### BOOKS RECEIVED.

T. FISHER UNWIN.

"Names and Their Meaning. A Book for the Curious." By Leopold Wagner.

"'93, or The Revolution Amongst the Flowers." By Florence Byng.

"Another Brownie Book." By Palmer Cox.

"Daddy Jake. The Runaway, and Short Stories told after Dark." By Uncle Remus.

J. AND A. CHURCHILL.

"A Short Manual of Orthopædy. By Heather Bigg, F.R.C.S. Part I.

### The Student of Medicine.

### DEATH OF A BRISTOL STUDENT.

It is with great regret we have to record the death of Mr. W. A. Price, age twenty-four, a senior student of the Bristol Medical School. The circumstances connected with his death form one of the sad aspects of student life, which, fortunately rare, are not altogether preventable. Mr. Price was acting as dresser to a patient in a surgical ward suffering from blood poisoning and in some way became inoculated. He first began to complain of feeling unwell and of slight redness of the seat of inoculation on November 28th; by December 8 his condition gave rise to the greatest anxiety, and, becoming rapidly worse, he sank and died on the 15th inst. Mr. Price was of a bright and genial nature, and his untimely end is deeply felt by the medical staff, his fellow students, and all connected with the Royal Infirmary and Medical School. A special service was held on the 17th inst., in the chapel of the Royal Infirmary, by the Rev. C. Goodall, chaplain, at which most of the members of the staff, the resident officers, students, and nurses were present. He was buried at Llantwit, near Brigend, on December 19th.

### APPOINTMENTS.

Barrett, R. H., L.R.C.P..Lond., M.R.C.S., re-appointed Medical Officer for the Second and Third B. Sanitary Districts of the Wisbech Union. Campbell, Dr., appointed Medical Officer for the Usk District of the Pontypool Union. Campbell, John, F.R.C.S.Eng., M.A., M.D., M.Ch., M.A.O.I., appointed Surgeon to the Samaritan Hospital for Women, Belfast. Copley, W. H., L.R.C.P.Lond., M.R.C.S., re-appointed Medical Officer for the Seventh Sanitary District of the Wisbech Union. Cort, John G. D., L.R.C.P.Lond., M.R.C.S.Eng., appointed Medical Officer for the Second District of the Blackburn Union. Craig, J., M.D., B.Ch., M.R.C.P.I., appointed Surgeon to the Meath Hospital, Dublin. Craigie, James, M.D., C.M., B.Sc., Public Health, Edin., appointed Medical Officer of Health for the Burgh of Musselburgh, N.B. Culross, James, M.A., M.B., C.M., appointed Medical Officer and Public Vaccinator to the Newton Abbot (No. 4) District. Douglas, Norman G., L.R.C.P., L.R.C.S.Edin., appointed Medical Officer to the Western District of the Scarborough Union, and Public Vaccinator for the Borough. Evans, Edward Prichard, L.R.C.P.Edin., M.R.C.S.Eng., re-appointed Medical Officer of Health for the Mountain Ash Urban Sanitary District. Fraser, H. E., M.A.Edin., M.B., C.M.Edin., appointed Resident Medical Officer to the Northern Infirmary, Inverness. Gilbert, H. P., L.R.C.P., Edin., M.R.C.S., re-appointed Medical Officer for the Eleventh Sanitary District of the Wisbech Union. Groom, William, jun., M.D.Camb., M.R.C.S., re-appointed Medical Officer for the Newton and Tydd St. Giles District of the Wisbech Union. Guisani, Joseph, M.D., R.U.I., M.Ch., appointed Visiting Physician to the Cork Union Hospital. Hall, J. Moore, M.D., B.Ch., B.A.O., Royal University Ireland, M.R.C.S., D.P.H.Eng., appointed Senior House Surgeon. Hughes, Edward, L.R.C.P., L.R.C.S.Edin., appointed Medical Officer for the Collieries at Ton, Ystrad, Rhondda. Jones, Robert, M.D.Lond., B.S., F.R.C.S.Eng., L.R.C.P.Lond., L.S.A., appointed Medical Superintendent to the London County Council's New Asylum at Claybury. Kempe, Arthur Wightman, M.D.Brux., M.R.C.P.Edin., re-appointed Medical Officer of Health to the Budleigh Local Board. Keyworth, Arthur F., M.R.C.S., L.R.C.P.I., appointed Surgeon to the Marple District of the Cheshire County Police. Knowsley Sibley, W., M.A., M.D., B.C.Cantab., M.R.C.S.Lond., appointed Clinical Assistant to the Skin Department, Middlesex. Kynoch, John A. C., M.B., C.M.Edin., appointed *pro tem.* Medical Superintendent to the Dundee Royal Infirmary. Lloyd, Evan, M.B., C.M.Glas., re-appointed Medical Officer of Health for the Rural Sanitary District of the Tregaron Union. Mackay, William, L.R.C.P.Edin., L.M., L.F.P.S.Glas., re-appointed Medical Officer of Health for the Norton District of the Malton Union. Marshall, R. J., M.B., C.M.Glas., appointed Deputy Medical Officer for the Iver Sanitary District of the Eton Union. Mason, George, M.R.C.S., re-appointed Medical Officer for the Ninth Sanitary District of the Wisbech Union. Masters, L., L.D.S.Edin., appointed Honorary Dentist. Metcalfe, Dr., appointed Medical Officer for the Capel District of the Tonbridge Union. Milligan, William, M.D., C.M., appointed Lecturer on Diseases of the Ear, at the Owens College, Manchester. Muspratt, Charles Drummond, M.D., B.S.Lond., F.R.C.S.Eng., appointed Medical Officer for Out-patients to the Royal Victoria Hospital, Bournemouth. Nightingale, J., M.B.Edin., appointed Medical Officer of Health to the Horsforth Urban Sanitary Authority.

### The Hospital Advertiser.

### MEDICAL APPOINTMENTS.

**NORTH STAFFORDSHIRE INFIRMARY AND EYE HOSPITAL.**

Salary £80 per annum, with Board, Lodging, and Washing.

**THE GENERAL HOSPITAL, BIRMINGHAM.**

### THE STUDENT OF MEDICINE—(*continued*).

### VACANCIES.

#### Resident Medical Officers.

Birmingham and Midland Eye Hospital. Assistant House Surgeon. Salary £50 per annum, with apartments and board. Applications to the Chairman of the Medical Board by January 2nd, 1893.

Brecon Infirmary, 6, Bulwark, Brecon, South Wales. Resident House Surgeon, unmarried, doubly qualified. Salary £70 per annum, with furnished apartments, board, attendance, fire, and gas. Applications to W. Powell Price, Secretary, by December 26th.

Bridgwater Infirmary. House Surgeon. Salary £80 per annum, with board and residence. Applications to Mr. John Coombs, Honorary Secretary, by January 1st, 1893.

Central London Ophthalmic Hospital, Gray's Inn Road, W.C. House Surgeon. Rooms, coal, and light provided. Applications to the Secretary, by January 10th, 1893.

Chichester Infirmary, Chichester. House Surgeon, Salary £80 per annum, with board, lodging, and washing. Applications to E. E. Street, Secretary, by March 1st, 1893.

Croydon General Hospital. House Surgeon, doubly qualified, unmarried. Salary £100 per annum, increasing £100 yearly to £120, with board and residence. Applications to the Secretary by December 30th.

General Hospital, Birmingham. Assistant House Surgeon. Board, residence, and washing provided. Applications to the House Governor by December 31st.

Hull Royal Infirmary. Junior Assistant House Surgeon. Salary £40 per annum, with board and lodging, Applications to Chairman, House Committee, by December 26th.

Killarney District Lunatic Asylum. Assistant Medical Officer. Salary £100 per annum, with allowances valued at £100 yearly. Candidates must be unmarried, and not over 30. Applications to Dr. L. T. Griffin, Resident Medical Superintendent. Election on January 19th, 1893.

Manchester Royal Infirmary. Resident Medical Officer for the Fever Hospital at Monsall; not less than 25 years of age, doubly qualified. Salary £250 per annum, with board and residence. Applications to the Chairman of the Board by January 7th, 1893.

National Hospital for the Paralysed and Epileptic (Albert Memorial), Queen Square, Bloomsbury, W.C. House Physician, doubly qualified. Salary £100 per annum, with board and apartments. Applications to B. Burford Rawlings, Secretary and General Director, by January 5th, 1893.

North Staffordshire Infirmary and Eye Hospital, Hartshill, Stoke-on-Trent. House Surgeon; doubly qualified. Salary £120 per annum, increasing £10 yearly, with furnished apartments, board, and washing. Applications to the Secretary by January 21st, 1893

Sheffield Public Hospital and Dispensary. Senior Assistant House Surgeon. Salary £72 per annum, with board, lodging, and washing. Applications to Dr. Crochley Clapham, Honorary Secretary Medical Staff, The Grange, near Rotherham, by January 2nd, 1893.

Southport Infirmary. House Surgeon, doubly qualified. Salary £100 per annum, with board, furnished rooms, and attendance. Applications to Joseph Worall, Infirmary, Southport, by January 4th, 1893.

Stockport Infirmary. Junior House Surgeon. Appointment for six months. Board and residence provided, and £10 honorarium after six months' satisfactory service. Applications to Lieutenant-Colonel S. W. Wilkinson, Honorary Secretary, by December 27th.

West Herts Infirmary, Hemel Hempstead. House Surgeon and Dispenser, doubly qualified, unmarried. Salary £100 per annum, with board, furnished rooms, light, fire, attendance, and washing. Applications to the Secretary by January 31st, 1893.

#### Non-Resident Medical Officers.

Chelsea Hospital for Women, Fulham Road, S.W. Clinical Assistant. Fee £3 3s. for three months. Applications to the Secretary.

County of Chester. Medical Officer of Health, doubly qualified. Salary £100 per annum, and out-of-pocket travelling expenses. Applications to Reginald Potts, Clerk to the Cheshire County Council, Chester, by December 31st.

Hospital for St. Peter Port, Guernsey. Surgeon. Salary £50 per annum. Applications to the President of the Poor Law Board by December 29th.

Hostel of St. Luke, Devonshire Street, W. Honorary Visiting Medical Officer. Applications to the Secretary, Hostel of St. Luke, Church House, Westminster, by January 7th, 1893.

King's College, London. Medical Registrar. Applications to J. W. Cunningham, Secretary, by December 30th.

Royal Westminster Ophthalmic Hospital, King William Street, West Strand. Clinical Assistants. Applications to the Secretary, by December 31st.

Stratford-on-Avon Rural Sanitary Authority. Medical Officer of Health. Salary £110 per annum. Applications to John Charles Warden, 9, Guild Street, Stratford-on-Avon, by January 21st, 1893.

Stratford-on-Avon Urban Sanitary Authority. Medical Officer of Health. Salary £40 per annum. Applications to Thomas Hunt, Town Clerk, Municipal Office, Stratford-on-Avon, by January 2nd, 1893.

University College of South Wales and Monmouthshire, Cardiff. A Professor of Anatomy, and a Professor of Physiology. Stipend in each case £350 per annum. Applications to Ivor James, Registrar, by February 10th, 1893,

University of Edinburgh. Examiner in each of the following subjects: (1) Anatomy, (2) Chemistry and Laboratory Work for the First B.Sc. Examination in Public Health, (3) Midwifery, (4) Practice of Physic, and (5) Botany. The salary in each of the departments Nos. 1, 3, 4, and 5 is £75 a year, and in No. 2 £90 a year, with an allowance of £10 per annum for travelling and other expenses in the case of Examiners not resident in Edinburgh or the immediate neighbourhood. Applications to M. C. Taylor, Secretary, by January 9th, 1893.

University of Glasgow. Assistant Examiner in Zoology. Salary £30 per annum. Applications to the Secretary of the Court, Mr. Alan E. Clapperton, West Regent Street, Glasgow, by January 10th, 1893.

### The Hospital Advertiser.

### MEDICAL APPOINTMENTS—(*Continued*).

**HOUSE PHYSICIAN.**

Resident Medical Superintendent.

